# Quantitative evaluation of the canalis sinuosus relative to adjacent structures in cone-beam computed tomography images

**DOI:** 10.34172/japid.2024.014

**Published:** 2024-07-31

**Authors:** Sahar Jabali, Sajjad Pishva, Roghieh Bardal, Farough Bahrami, Maryam Mostafavi

**Affiliations:** ^1^Department of Periodontology, School of Dentistry, Urmia University of Medical Sciences, Urmia, Iran; ^2^Department of Oral and Maxillofacial Radiology, Dental Caries Prevention Research Center, Qazvin University of Medical Sciences, Qazvin, Iran; ^3^Department of Endodontics, School of Dentistry, Isfahan University of Medical Sciences, Isfahan, Iran; ^4^Department of Oral and Maxillofacial Radiology, School of Dentistry, Urmia University of Medical Sciences, Urmia, Iran

**Keywords:** Anatomic variations, Cone-beam computed tomography, Maxillary nerve

## Abstract

**Background.:**

Careful anatomical investigation of canalis sinuosus (CS) is essential to prevent damage to blood vessels and nerves in this area during surgical procedures, such as placing dental implants in the anterior maxillary region. This study investigated the relationship and distance between the CS and its adjacent structures.

**Methods.:**

A total of 400 cone-beam computed tomography (CBCT) images of Iranian adults aged 20–86 years were included in this retrospective study. Two observers assessed all the images twice with a time interval of one month. The closest tooth to the CS, its position relative to the CS, and distance measurements of the CS from adjacent structures were determined.

**Results.:**

CS was found in 10.5% of all images. The mean diameter of the canal was 1.06±0.29 mm, which was not significantly different between the age groups, right and left sides, or genders. The most common location of CS was mid-position relative to the upper lateral incisors. In linear measurements, only the distance from the CS to the buccal cortical plate and perpendicular to the nasopalatine canal exhibited a significant difference between the two sexes, with no significant difference between the right and left sides.

**Conclusion.:**

CS location was significantly more palato-lateral in males. There was no significant difference in the prevalence between the two sexes.

## Introduction

 The anterior region of the maxilla undergoes many surgical interventions. Dental implant placement, surgeries of supernumerary impacted teeth and cysts, and orthognathic surgeries are some of these interventions in the anterior maxilla.^1^ However, the most important of all these is the increasing demand for dental implants. Thus, a more precise anatomical investigation of this segment is essential due to the presence of canalis sinuosus (CS) in the anterior maxilla.^2^ CS is a neurovascular canal about 2 mm in diameter, which carries a branch of nerves of the infraorbital canal, the anterior superior alveolar nerve (ASA), and related vessels.^3,4^ The infraorbital nerve is a branch of the maxillary nerve, which is the second branch of the fifth cranial nerve, i.e., the trigeminal nerve. The skin distribution of the infraorbital nerve extends to the upper lip, cheeks, lower eyelids, outer nose, and nasal cavity.^5^

 On cone-beam computed tomography (CBCT) images, CS is a curved bone canal originating laterally from the infraorbital canal. It passes through an internal and anterior course to reach the maxillary anterior region, passes through the lateral wall of the nose, and is placed in the marginal part of the nasal cavity floor; then, lateral canals branch off, eventually opening next to the incisor canal in the palate.^6^ CS detection is important because damage to such structures might cause sensory disorders. In addition, such structures may be mistaken for other anatomical structures or lesions, leading to unnecessary or incorrect procedures.^7^ Therefore, accurately identifying the anatomy of the face, mouth, and jaws and using radiographic images, specifically CBCT in surgical procedures, are necessary to avoid the destruction of blood vessels and nerves in this area.^8^

 The anatomy of the CS^9-13^ and its accessory canals^14-19^ has been evaluated in numerous studies. A systematic review study conducted in 2023 considered CS and its accessory canals as anatomic structures due to their high prevalence.^20^ However, a few studies have quantitatively evaluated the canal’s relationship with adjacent structures.^4^ Therefore, this study investigated the exact position of CS relative to adjacent teeth and its distance from adjacent anatomical structures in CBCT images of an Iranian population.

## Methods

 The present retrospective study was conducted on 400 CBCT images of the anterior maxilla of Iranian patients referred to a private dental and maxillofacial radiology center in Urmia, Iran.

*The inclusion criteria:* (1) a chronological age of 12−90, (2) no history of systemic disease due to osteoporosis, (3) and good diagnostic quality of images.

*The exclusion criteria:* the images of edentulous patients and patients with dentoalveolar fractures, pathologic conditions, dental implants, or bone grafting in the anterior maxilla.

 The CBCT images were captured using an 8-cm field of view of Planmeca Promax 3D mid (Helsinki, Finland) with the following conditions: voxel size: 200 μm; time: 12 s; mA: 10; kVp: 90.

**Figure 1:**
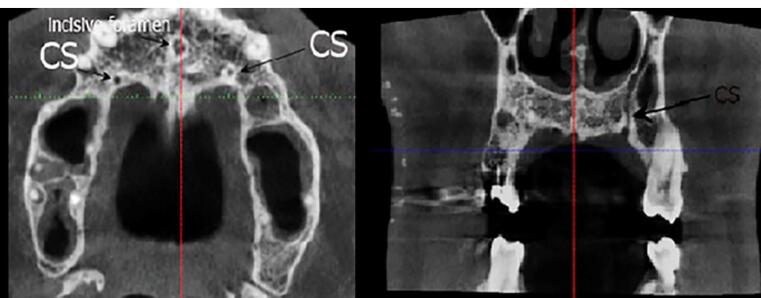
Axial and coronal views of the CS

**Figure 2:**
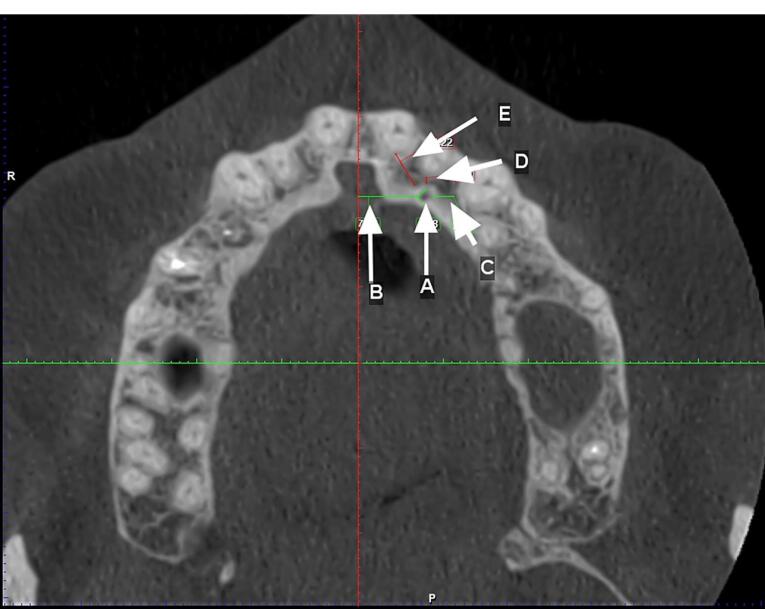
Linear measurements made in the axial plane: A, the CS diameter; B, the CS distance from the perpendicular to the nasopalatine canal; C, the CS distance from the distal tooth; D, the CS distance from the main tooth; E, the CS distance from the mesial toot

**Figure 3:**
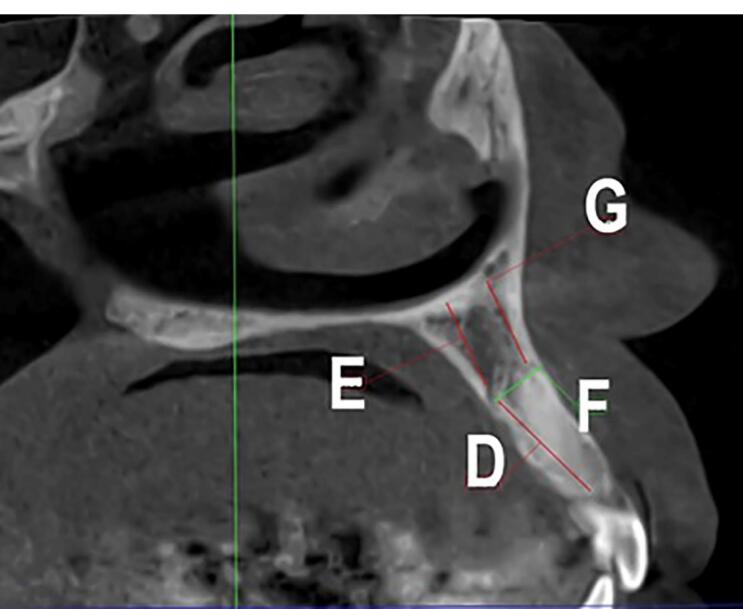
Linear measurements made in the cross-section: D, the CS distance from the crest ridge; E, the CS distance from the nasal cavity floor; F, the CS distance from the buccal cortical plate; G, the distance of canal extension from the main tooth apex

 Two observers investigated all the images twice with a time interval of one month. The first observer was an experienced periodontist, and the second observer was an experienced oral and maxillofacial radiologist. Observations and measurements were carried out by Planmeca Romexis software version 3.8.1. The presence of CS was defined in axial and sagittal planes, and its clear extension was confirmed in the coronal sections. CS diameter was measured on the axial plane. Canals with a diameter of > 1 mm and a definite extension to the infraorbital canal were considered (Figure 1). Slice thickness and interval were 0.5 in all the sections. The axial plane in which the CS had the largest diameter was chosen to measure the distance of the CS to the adjacent teeth and the nasopalatine canal. The tooth with the closest distance to the CS was considered the main tooth; then, the mesial, mid, or distal position of the main tooth related to the CS was determined. The distances of CS from the nasal floor, ridge crest, buccal cortical plate, and the distance of canal extension from the main tooth apex were measured on the cross-sections perpendicular to the axial plane at the CS site (Figures 2 and 3).

 Intra- and inter-observer reliability was evaluated in 10% of the CBCT images after two weeks using the intraclass correlation coefficient (ICC).

###  Statistical analysis 

 The data were analyzed with SPSS 22 using descriptive statistics, i.e., maximum, minimum, mean, and standard deviation. The ICC value was ˃0.80 for both intra- and inter-observer reliabilities. Pearson’s correlation coefficient was used to determine the relation between two quantitative variables. Fisher’s exact test was used to compare CS distribution in terms of sex and location. T-test was used to compare distance measurements between males and females or between the right and left sides. *P* ≥ 0.05 was considered statistically insignificant.

## Results

 CBCT images of 400 patients were investigated, with 185 males (46.3%) and 215 (53.7%) females. The mean age of the patients was 43.06 ± 13.60, with a range of 20–86 years. Forty-two patients (10.5%) had at least one CS with a clear extension towards the infraorbital canal, with a diameter of > 1 mm; 20 (47.6%) were female, and 22 (53.4%) were male. In 13 patients (30.95%), the canal was bilateral, and in 29 (69.05%), the canal was unilateral. In 25 (86.2%) patients, the canal was on the right side, and in 4 (13.80%), the canal was on the left side; generally, 55 canals were observed in images.

Table 1 shows the mean diameters of CS in both sexes. There was no significant difference in the frequency of cases between the two genders (*P* > 0.05). No significant correlation was observed between CS diameter and age (*P* = 0.101) or sex (*P* = 0.284). CS was more frequent on the right side than on the left side, but this difference was not significant (*P* > 0.05) (Table 2). The CS had the closest distance to the lateral incisor in most cases. The mid-position of the CS compared to the main tooth was significantly more common than other positions (*P* = 0.04).

**Table 1:** CS diameter in mm according to sex

**Sex**	**Number**	**Mean (SD)**	**Minimum**	**Maximum**
Male	28	1.10 (0.31)	0.67	1.75
Female	27	1.02 (0.28)	0.75	1.81
Total	55	1.06 (0.29)	0.60	1.81

CS:*canalis sinuosus*

**Table 2:** Frequencies (%) of CS in the patients according to sex, side, and position

	**Sex**		**Side**		**Position**		
	**Male (%)**	**Female (%)**	**Right (%)**	**Left (%)**	**Mesial-position (%)**	**Mid-position (%)**	**Distal-position (%)**
Central Incisor	1 (1.8)	2 (3.6)	2 (3.6)	1 (1.8)	0 (0.0)	1 (1.8)	2 (3.6)
Lateral Incisor	12 (21.8)	17 (30.9)	18 (32.7)	11 (20)	4 (7.3)	20 (36.3)	5 (9.1)
Canine	11 (20)	7 (12.7)	13 (23.6)	5 (9.1)	1 (1.8)	8 (14.6)	9 (16.5)
First Premolar	4 (7.3)	1 (1.8)	5 (9.1)	0 (0.0)	2 (3.6)	1 (1.8)	2 (3.6)
Total	28 (50.9)	27 (49.1)	38 (69.1)	17 (30.9)	7 (12.7)	30 (54.5)	18 (32.8)
*P* value	0.32		0.41		0.04		

CS:*canalis sinuosus.*

 The average distances of CS from the nasal floor, ridge crest, buccal cortical plate, main tooth apex, and nasopalatine canal were higher in males than in females; this difference was not statistically significant except for the buccal cortical plate distance and perpendicular distance to the nasopalatine canal (Table 3). The distance of CS from neighboring structures on the right and left sides was not statistically significant (*P* > 0.05).

**Table 3:** Means and standard deviations (SDs) of the distances between CS and anatomic landmarks according to sex and side

**Anatomic landmark**	**Sex**		***P*** ** value**	**Side**		***P*** ** value**	**Total**
	**Male**	**Female**		**Right**	**Left**		
Nasal cavity	11.58 (3.63)	10.17(3.61)	0.15	11.24 (3.48)	10.66(3.80)	0.58	10.89 (3.66)
Buccal cortical	8.92 (1.58)	8.10 (0.98)	0.02	8.46 (1.29)	8.56 (1.44)	0.80	8.52 (1.37)
Ridge crest	10.48 (4.13)	9.30 (2.81)	0.22	9.34 (3.10)	10.27 (3.84)	0.34	9.90 (3.56)
Main tooth apex	4.60 (2.31)	4.21 (1.28)	0.44	4.58 (1.51)	4.30 (2.09)	0.57	4.41 (1.87)
Main tooth	2.19 (1.88)	1.66 (0.67)	0.16	1.83 (1.20)	2.00 (1.58)	0.66	1.93 (1.43)
Distal tooth	5.28 (1.30)	4.72 (1.70)	0.17	5.32 (1.58)	4.80 (1.49)	0.25	4.99 (1.53)
Mesial tooth	4.96 (1.70)	4.52 (1.23)	0.27	4.68 (1.16)	4.77 (1.67)	0.81	4.73 (1.48)
Perpendicular to the nasopalatine canal	9.48 (3.19)	7.26 (2.20)	0.00	9.14 (3.40)	7.90 (2.54)	0.18	8.39 (2.95)

CS:*canalis sinuosus.*

## Discussion

 CS is a branch of the infraorbital nerve, which is the maxillary division of the trigeminal nerve. Damage to this canal during surgery can lead to complications such as bleeding and paresthesia. CS can be detected in special imaging modalities such as thin sections of CBCT.^4^

 This study evaluated the location and distance of CS relative to adjacent structures in the Iranian population because of anatomical variations between different populations.

 The prevalence of CS in this study was 10%, which was close to a study by de Oliveira-Santos et al. (15.7%)^21^; this prevalence was about 35% in a study by Manhães Júnior et al,^4^ 66.5% in a study by Aoki et al,^13^ 88% in a study by Wanzeler et al,^5^ and 98.5% in a study by Yeap et al.^22^ However, the prevalence of CS in a study by Gurler et al^23^ was 100%. The possible reasons for this discrepancy include differences in the slice thickness of the CBCT images,^23^ the software used, sample size, and the content of studies.^11^ In addition, in this study, cases traceable to the infraorbital canal with a diameter of > 1 mm were selected, which could be another reason for the difference.

 The canal was bilateral in 13 patients (30.95%) of the 42 patients with CS. The frequency percentage of bilateral cases of CS was various in other literature as follows: de Oliveira-Santos et al.^21^ reported 21.4%, Manhães et al^4^ reported 24.3%, Aoki et al^13^ reported 54.1%, and Gurler et al^23^ reported 100%.

 There was no significant difference in the overall frequency of CS on both sides (*P* = 0.41), consistent with the Manhães et al. study.

 Gender distribution of CS was not statistically significant, consistent with studies by Gurler et al,^23^ Von Arks et al,^6^ Machado et al,^19^ and Wanzeler et al.^5^ In a study by Anatoly et al,^24^ the prevalence of CS was significantly higher in females, while this prevalence was significantly higher in males in Aoki et al. study.^13^

 The mean (SD) diameter of CS was 1.06 (0.26) mm, which was significantly (*P* = 0.284) higher in males [mean (SD) diameter in males = 1.10 (0.31) vs. females = 1.02 (0.28)]. In the study by Aoki et al,^13^ this difference was not significant, either. Gurler et al.^23^ reported a significantly higher mean diameter of CS in males (*P* = 0.001).

 Because of the difficulty of some cases in precisely attributing the CS to the specific tooth,^16^ Beyzade et al^25^ modified the classification used by Oliveira-Santos et al.^21^ For the same reason, in this study, CS’s distance to the neighboring teeth was determined in axial plane in which the CS had the most diameter, at first. The position of the CS with regard to the closest tooth was determined at the second. CS was most related to the mid-position of lateral incisors. CS was more commonly associated with this tooth in other populations.^4,11,22,24^

 In this study, CS-to-the-buccal cortical plate and CS-perpendicular-to-nasopalatine canal distances were significantly higher in males, so the CS location was more palato-lateral. In the Manhães Júnior et al^4^ study, the nasal cavity distance was higher than in our study, and the ridge crest and the buccal cortical plate distances were lower than in our study, so the CS was located in a more buccal position and closer to the crest in Manhães Júnior and colleagues’ study.

## Conclusion

 The prevalence, diameter, and most of the linear distances of the CS to the adjacent structures were not significantly different between the two genders. However, CS had a significantly more palato-lateral position in males. There were variations in the prevalence, location, and linear distances between different populations.

## Acknowledgments

 The authors would like to thank Dr. Majid Abdolrahimi for his cooperation in writing the manuscript.

## Competing Interests

 The authors declare that they have no competing interests.

## Consent for Publication

 Not applicable.

## Data Availability Statement

 The data that support our results are available upon reasonable request from the corresponding author, Maryam Mostafavi.

## Ethical Approval

 This study was approved by the Ethics Committee of Urmia University of Medical Sciences with the ethical number of IR.UMSU.REC.1397.064. There was no conflict with ethical considerations.
